# Pharmacoeconomic implications of preference toward reference- versus generic-brand antidepressants in primary care

**DOI:** 10.1017/S1463423624000276

**Published:** 2024-09-20

**Authors:** Onur Gultekin, Volkan Aydin, Dilara Bayram, Omer Atac, Ahmet Akici

**Affiliations:** 1 Department of Medical Pharmacology, School of Medicine, Marmara University, Istanbul, Turkey; 2 Department of Medical Pharmacology, International School of Medicine, Istanbul Medipol University, Istanbul, Turkey; 3 Department of Pharmacology, School of Pharmacy, Acibadem Mehmet Ali Aydinlar University, Istanbul, Turkey; 4 Department of Public Health, International School of Medicine, Istanbul Medipol University, Istanbul, Turkey; 5 Department of Health Management and Policy, College of Public Health, University of Kentucky, Lexington, KY, USA; 6 Department of Medical Pharmacology, School of Medicine, Eastern Mediterranean University, Famagusta, North Cyprus

**Keywords:** antidepressive agents, drug utilization, generic drugs, pharmacoeconomics, primary health care

## Abstract

**Background:**

The prevalence of depression is gradually increasing worldwide with an increasing utilization of antidepressants. Nevertheless, despite their lower costs, generic-brand antidepressants were reported to be less prescribed. We aimed to examine the costs of reference- versus generic-brand antidepressant prescriptions in primary care practice.

**Methods:**

This cross-sectional study included electronic prescriptions for adult patients that contained antidepressants (World Health Organization’s Anatomical Therapeutic Chemical (ATC) code: N06A), which were generated by a systematically selected sample of primary care doctors (*n* = 1431) in Istanbul in 2016. We examined the drug groups preferred, the reference- versus generic-brand status, and pharmacotherapy costs.

**Findings:**

The majority of the prescriptions were prescribed for women (71.8%), and the average age of the patients was 53.6 ± 16.2 years. In prescriptions with a depression-related indication (*n* = 40 497), the mean number and cost of drugs were 1.5 ± 1.0 and 22.7 ± 26.4 United States Dollar ($) per prescription, respectively. In these prescriptions, the mean number and cost of antidepressants per encounter were 1.1 ± 0.2 and $17.0 ± 13.2, respectively. Reference-brand antidepressants were preferred in 58.2% of depression-related prescriptions, where the mean cost per prescription was $18.3 ± 12.4. The mean cost per prescription of the generics, which constituted 41.8% of the antidepressants in prescriptions, was $15.1 ± 11.4. We found that if the generic version with the lowest cost was prescribed instead of the reference-brand, the mean cost per prescription would be $12.9 ± 11.2.

**Conclusions:**

Our study highlighted the substantial pharmacoeconomic impact of generic-brand antidepressant prescribing, whose preference over reference-brands could reduce the cost of antidepressant medication treatment by 17.5% in primary care, which could be approximately doubled if the cheapest generic antidepressant had been prescribed.

## Introduction

Depression is among the leading reasons for disability worldwide and its prevalence has a markedly increasing trend in the last decades (Institute for Health Metrics and Evaluation, 2019; Moreno-Agostino *et al.*, 2021). The main pharmacological approach to manage depression is to use antidepressants such as selective serotonin reuptake inhibitors (SSRIs), serotonin/noradrenaline reuptake inhibitors (SNRIs), and tricyclic antidepressants (TCAs) (American Psychiatric Association, 2013). An Organisation for Economic Co-operation and Development (OECD) report revealed that the consumption of antidepressant drugs doubled in member countries between 2000 and 2017 (OECD, 2019). Another study reported the number of antidepressants used in Turkey in 2012 to reach 37.4 million boxes, with a 2.6-fold increase than that in 2003 (Aydin *et al.*, 2013).

The term “generic drug” refers to a pharmaceutical product that is typically interchangeable with the first-approved brand drug and marketed by another company after the patent or other exclusive rights have expired. Contrary to the common misconception, a generic medication shares all the same properties as its equivalent reference drug, including efficacy, safety, quality, dosage form, strength, administration method, and indication (Hassali *et al.*, 2014). According to a 2019 report from the USA, generic-brand drugs accounted for about 90% of all dispensed products and contributed to save $293 billion in healthcare costs (Association for Accessible Medicines, 2019). In addition to the projected $450 billion annual cost of prescription drugs in the USA, many essential pharmaceutical treatments for chronic illnesses, including depression, were reported to be purchased for $4 per month or less in 2017 owing to an expanding generic drug industry (Liu *et al.,* 2018; Kesselheim *et al.,* 2019).

Primary care physicians were reported to prescribe over half of the antidepressant drugs in registered prescriptions between 2008 and 2017 in Turkey (Yalçın and Öztürk, 2016). Primary care institutions are central to the functioning of primary health care, as they are easily accessible and constitute the first level of contact as well as for people seeking help for mental health problems (John *et al.*, 2016). It makes sense that psychiatry-related prescriptions issued in primary care could provide reliable and valuable information about health indicators. We aimed to examine the costs of reference- versus generic-brand antidepressant prescriptions in primary care practice.

## Method

In this cross-sectional study, we examined the prescriptions registered to the National Prescription Information System and issued in primary care centers in Istanbul in 2016. The city had a population of 14.6 million in 2016 and was inhabited by 17.9% of primary care physicians in the country (Turkish Statistical Institute, 2017). The study was approved by the Ethics Committee for Non-Interventional Clinical Research of Istanbul Medipol University (Approval No: 13/10/2022-865).

**Figure 1.**
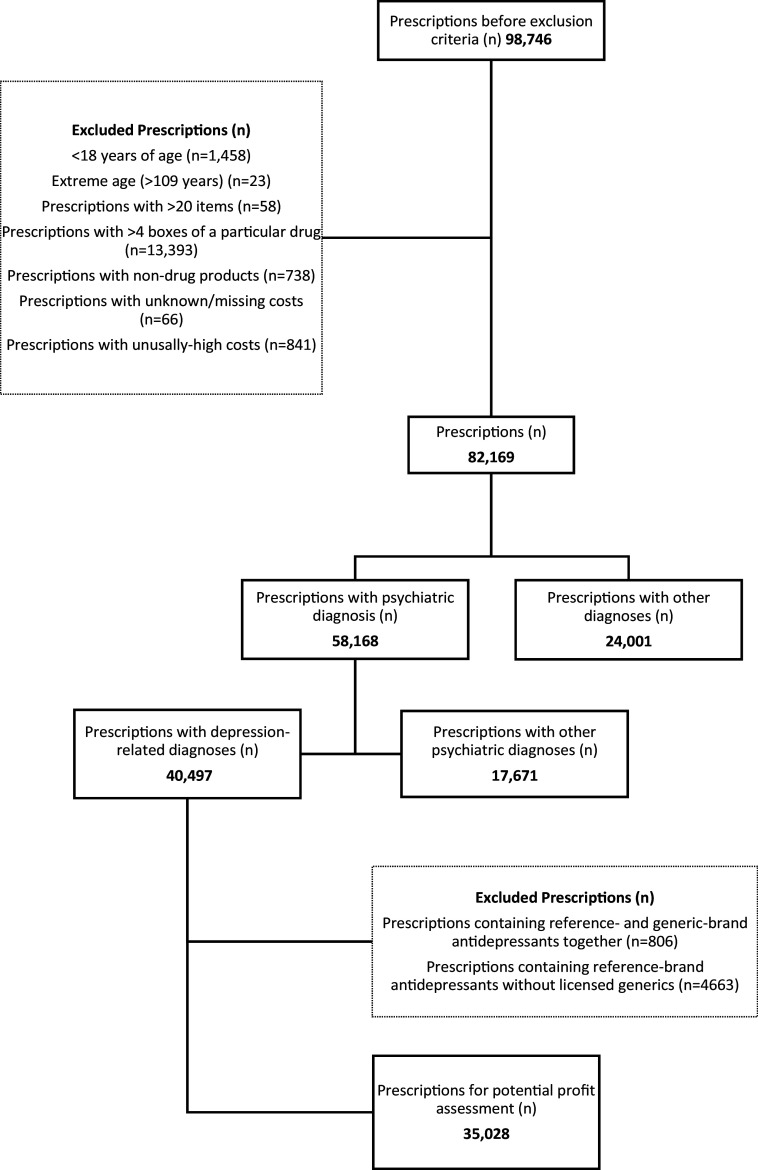
Flowchart of the prescriptions included in the study.

We performed a 3:1 systematic sampling of all primary care physicians serving in Istanbul in 2016. Of the resultant 1431 physicians, we selected the prescriptions for at least one antidepressant medication and single indication (*n* = 98 746). We excluded the prescriptions for children (<18-year-old), those containing >20 different drugs or >4 packs of a particular drug (very likely to be an erroneous prescribing incident), non-drug products and those with unknown, missing, or unusually high costs (>1000 Turkish liras [TL], equivalent to 340.1 US dollars [$]) (Figure 1). In these antidepressant-containing prescriptions (ACP, *n* = 82 169), we determined demographic characteristics including age groups (“18–65 years” and “≥65 years”) and gender, number of drugs per prescription, distribution of diagnoses in prescriptions, and their costs. We categorized prescriptions by their diagnosis as psychiatric (International Classification of Diseases (ICD) main code: F) and nonpsychiatric and further evaluated those with a diagnosis of depression (ICD code: F20.4, F31.3, F31.4, F31.5, F32–F33, F34.1, F41.2, F92.0) (*n* = 40 497). We determined the reference- and generic-brand status of the antidepressant drugs in these prescriptions.

### Cost assessments

We calculated the costs of antidepressant drugs by their reference-/generic-brand status. The cost of the prescriptions in TL was exchanged to $ based on the mean parity rate in 2016. Prescriptions containing reference- and generic-brand antidepressants together or a reference-brand antidepressant that had no commercially available generic in the market were excluded from the comparative cost analysis. The potential profit calculation was based on the scenario of prescribing the cheapest brand on the market instead of the reference-brand antidepressant. During the cost simulation, the cheapest generic-brand antidepressant available in the database with a listed price was preferred over the reference-brand antidepressant. The quantity of the generic-brand drug required to match the dosage and number of tablets of the reference-brand drug was calculated.

### Statistical analysis

Statistical analyses were performed using IBM SPSS Statistics 22.0 (IBM Corp., Armonk, NY, USA), Microsoft Excel 2021 for Windows (Microsoft Corp., Redmond, WA, USA), and GraphPad Prism 5.0 (GraphPad Software, San Diego, CA, USA) software. Results were expressed as numbers and percentages for categorical variables and mean and standard deviation for continuous variables. Chi-square analysis was used to compare the categorical variables of the groups. Continuous variables were tested for normality and compared via the t-test if normally distributed or via the Mann–Whitney U test if non-normally distributed. Overall, a 5% type-1 error level was assumed to be acceptable to infer statistical significance.

## Results

The mean age of the patients was 53.6 ± 16.2 years, and the prescriptions were mostly generated for women (71.8%). We identified 2.2 ± 1.7 drugs per prescription with a mean cost of $30.4 ± 36.2 per prescription (Supplementary Table 1). Nonpsychiatric diagnoses constituted 29.2% of the prescriptions, led by the circulatory system diseases (26.8%) as detailed in Supplementary Table 2. Psychiatric indications constituted 70.8% of the prescriptions with 1.5 ± 1.0 drugs per prescription. The mean cost in these prescriptions was $22.8 ± 28.6.

In prescriptions with a depression-related indication (*n* = 40 497), the mean number and cost of drugs per prescription were 1.5 ± 1.0 and $22.7 ± 26.4, respectively. In these prescriptions, the mean number and cost of antidepressants per prescription were 1.1 ± 0.2 and $17.0 ± 13.2, respectively. The most common diagnosis was a “single episode of unspecified major depressive disorder” (35.8%). We identified the highest cost per prescription for the “single episode of moderate major depressive disorder” ($29.9 ± 34.0) (Table 1). In depression-related prescriptions, SSRIs were the mostly preferred group (80.0%), followed by SNRIs (9.5%), 5-HT2 receptor modulators (3.2%), TCA (2.8%), and heterocyclic antidepressants (2.5%). We detected that all TCA and 5-HT2 receptor modulators were prescribed as the reference-brand, followed by SNRI (65.9%), SSRI (61.2%), and heterocyclic antidepressants (39.5%).

**Table 1.** The mean number of drugs and cost per prescription based on diagnosis groups

Diagnosis (ICD-10)	Prescription *n* (%)	NDPP	CPP ($)
Nonpsychiatric indications	24 001 (29.2)	3.8 ± 1.9	48.8 ± 45.1
Psychiatric indications	58 168 (70.8)	1.5 ± 1.0	22.8 ± 28.6
Non-depression psychiatric diagnoses	17 671 (30.4)	1.4 ± 0.9	23.0 ± 33.1
Depression-related diagnoses	40 497 (69.6)	1.5 ± 1.0	22.7 ± 26.4
Major depressive disorder (F32.9)	14 478 (35.8)	1.6 ± 1.1	23.1 ± 26.6
Depressive conduct disorder (F92.0)	8870 (21.9)	1.3 ± 0.7	20.2 ± 22.1
Other depressive episodes (F32.8)	6752 (16.7)	1.3 ± 0.7	20.6 ± 22.4
Depressive episode (F32)	3929 (9.7)	1.7 ± 1.4	24.9 ± 30.1
Mixed anxiety and depressive disorder (F41.2)	1977 (4.9)	1.6 ± 1.1	23.1 ± 27.7
Major depressive disorder, single episode, mild (F32.0)	1284 (3.2)	1.4 ± 0.9	21.6 ± 25.0
Major depressive disorder, single episode, moderate (F32.1)	1257 (3.1)	1.4 ± 0.8	29.9 ± 34.0
Major depressive disorder, recurrent, unspecified (F33.9)	729 (1.8)	1.5 ± 1.0	22.6 ± 25.5
Other recurrent depressive disorders (F33.8)	315 (0.8)	1.7 ± 1.3	27.3 ± 30.2
Major depressive disorder, recurrent (F33)	286 (0.7)	1.3 ± 0.8	21.5 ± 21.8
Other	620 (1.5)	1.7 ± 0.9	42.8 ± 50.0
Total	40 497 (100)	1.5 ± 1.0	22.7 ± 26.4

NDPP = number of drugs per prescription; CPP = average cost per prescription.

**Figure 2.**
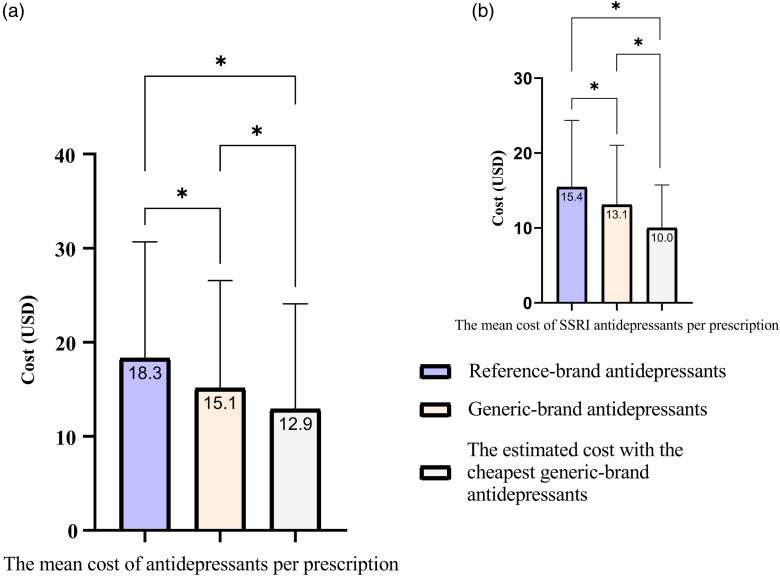
The mean cost of antidepressants per prescription with a depression-related diagnosis (*: *P* < 0.0001).

Among depression-related prescriptions containing either reference- or generic-brand antidepressants (*n* = 35 028), 58.2% had reference-brand antidepressants, and 41.8% had generic-brand antidepressants. The mean cost of the prescriptions with generic-brand antidepressants ($21.2 ± 26.2) was significantly lower than that of reference-brand antidepressants ($23.9 ± 25.7, *P* < 0.0001) and significantly higher than that of the assumed cheapest brand selection instead of the reference-brand ($19.6 ± 25.6, *P* < 0.0001). The mean cost of antidepressants in prescriptions with generic-brand antidepressants ($15.1 ± 11.4) was significantly lower than that of reference-brand antidepressants ($18.3 ± 12.4, *P* < 0.0001) and significantly higher than that of the assumed cheapest brand selection instead of the reference-brand ($12.9 ± 11.2, *P* < 0.0001, Figure 2a).

**Figure 3.**
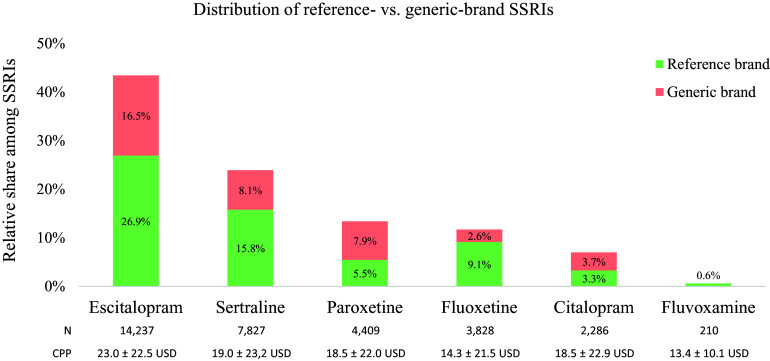
Distribution of SSRIs and reference- versus generic-brand prescribing status.

Depression-related prescriptions containing only SSRIs (*n* = 32 642) showed a preference for generic brands at 38.8%. The mean cost of antidepressants in prescriptions with generic-brand SSRIs ($13.1 ± 8.0) was significantly lower than that of reference-brand antidepressants ($15.4 ± 8.9, *P* < 0.0001) and significantly higher than that of the assumed cheapest brand preference instead of the reference-brand ($10.0 ± 5.7, *P* < 0.0001, Figure 2b). Among the most commonly prescribed antidepressants, SSRIs, escitalopram (43.4%) was found to be the most prescribed, followed by sertraline (23.9%), paroxetine (13.4%), and fluoxetine (11.7%). The highest rate of generic-brand preference was detected in paroxetine (59.2%), whereas the lowest rate of that was detected in fluoxetine (21.9%) (Figure 3).

## Discussion

Apart from evaluating the trend of generic-brand antidepressant prescribing, our study is the first to reveal the pharmacoeconomic impact of this situation in primary care prescription data in Turkey. Revealing an overprescribing of reference brands, our findings suggest the preference of generics over reference antidepressants could reduce antidepressant-related costs in primary care by 17.5%. Moreover, this cost-saving could be doubled if the cheapest alternative available in the market had been selected, especially in terms of SSRIs, the most preferred antidepressant group in this study. Since most prescribed antidepressants are reference-brand drugs, our assumption is likely to underscore the considerable pharmacoeconomic impact of selecting generic-brand antidepressants over their reference-brand counterparts.

We previously reported the share of generic drug prescribing as 54.0% in Turkey between 2013 and 2016, with as low as 41.4% for depression prescriptions (Bayram *et al.*, 2021). While this seemed to be consistent with our finding (41.8%), we observed it much lower for SSRIs (38.8%), the predominant group in depression pharmacotherapy. While mental health was reported to be one of four therapeutic areas accounting for the greatest savings afforded from generic drugs like antidepressants, various case reports and bioequivalence study results have led to debates that generic-brand antidepressants have disadvantages in efficacy and tolerability (Dunn *et al.*, 2006; Kautzner *et al.*, 2011; Association for Accessible Medicines, 2017). Furthermore, several case reports and studies describe clinical deterioration and reduced tolerability with generic substitution (Desmarais *et al.*, 2011). In fact, many psychiatrists reportedly expressed concerns about generic drugs (Cessak *et al.*, 2016). A survey conducted with psychiatrists in Germany reported that physicians preferred the reference-brand antidepressants for their own use more than the drugs they prescribe to their patients (Hamann *et al.*, 2013). It can be thought that these concerns may be among the reasons for the majority of reference-brand antidepressants in the treatment of depression in our study. On the other hand, a US study found no significant difference between those who started antidepressant treatment with the reference-brand and those who started with the generic-brand in terms of discontinuing the treatment (Dunn *et al.*, 2006). Therefore, considering its increasing frequency and share in terms of drug use, it can be considered one of the important intervention areas in increasing the use of generic-brand drugs in depression (Mojtabai and Olfson, 2014).

Generic-brand drugs were reported to account for 89% of all prescriptions in 2016 in the USA, although accounting for just 27% of total prescription costs (Hamann *et al.*, 2013). One of the primary reasons for this is that the retail price of a generic drug is on average 75% less than that of a reference drug (Congressional Budget Office, 2010). Only with the use of generic-brand drugs instead of reference brands, health insurance expenditures in the USA were reported to decline by $67.6 billion and healthcare expenditures by $32.7 billion in 2015, emphasizing the slowdown of mounting US healthcare costs by switching to generic brands (Howard *et al.*, 2018). Consistent with this improvement, if the cheapest generic-brand antidepressant were chosen instead of the current reference-brand among our study population, the average antidepressant cost would decrease from $18.3 to $12.9 with an approximately 30% reduction, indicating that the use of generic-brand drugs in this drug group has an important place in health expenditures (OECD, 2019; Basara *et al.*, 2019). In addition, considering that more than half of the antidepressant drugs are prescribed by primary care physicians in the country, this study contributed to uncover pharmacoeconomic reflections of the antidepressant prescribing patterns of primary care (Aydin *et al.*, 2013).

In our study, approximately one-third of prescriptions containing antidepressants were generated for non-psychiatric diagnoses. A Canadian study examining primary care prescriptions between 2006 and 2015 reported that 55% of prescriptions containing antidepressants were diagnosed with depression (Wong *et al.*, 2016). In our study, we found that approximately half (49.3%) of the ACPs included a diagnosis of depression. This situation can be considered as a serious problem of irrationality in terms of rational pharmacotherapy principles. Therefore, drug-diagnostic mismatches in prescriptions can cause negative effects on cost-related processes such as reimbursement processes, as well as many medical problems. This might be further compelled by another finding in our study that we observed a higher frequency of generic-brand prescribing as the number of drugs per encounter increased, suggestive of concomitant chronic conditions. Contrarily, the use of generic-brand drugs in patients with chronic diseases was reported to be lower than in patients with less chronic diseases (Hassali *et al.*, 2005; Himmel *et al.*, 2005). Therefore, it may be suggested that prescribing drugs for imprecise reasons with costly consequences could be regarded as another target for areas of development in raising awareness about the appropriate use of medicines.

Our study has some limitations. Receiving single-diagnosis prescriptions may lead to the elimination of various data; this allowed more specific and consistent inferences to be made in terms of diagnoses. On the other hand, the study provided primary care data, warranting additional evaluations of all health services. We used a comparably older data set, yet this is primarily because it was the most recent database we could access and since the coronavirus disease 2019 (COVID-19) pandemic has altered the prescribing routines of physicians, etc. We preferred to use the data set of a large metropolitan city that we could access in the pre-COVID-19 period so that differences such as these have little impact on our findings.

In conclusion, our study showed higher prescribing rates of reference-brand antidepressant drugs in depression pharmacotherapy in primary practice. Physicians who chose generics over reference-brand antidepressants in primary care could downshift the cost of antidepressant medication treatment by 17.5%, with a potential doubling of this cost-saving if the cheapest generic-brand antidepressant drugs had been preferred. This indicates the need for reemphasizing generic-brand drug awareness in depression pharmacotherapy with respect to cost-effective health expenditure measures. Moreover, the reproducibility of our study in other countries and the comparative assessment could further emphasize the global importance of adopting cost-effective health expenditure measures in depression pharmacotherapy in primary practice.

## Supporting information

Gultekin et al. supplementary material Gultekin et al. supplementary material

## Supplementary material

To view supplementary material for this article, please visit https://doi.org/10.1017/S1463423624000276.
