# Supplementary material for: Pharmacoeconomic implications of preference toward reference- versus generic-brand antidepressants in primary care
Source: Prim Health Care Res Dev. 2024 Sep 20;25:e40. doi: 10.1017/S1463423624000276 (PMC11464804; doi:10.1017/S1463423624000276)
Supplement: Gultekin et al. supplementary material [file S1463423624000276sup001.docx]

**Supplementary Table 1.** Distribution of ACPs by gender and age groups.

|  | | | **ACPs** |
| --- | --- | --- | --- |
| **Gender** | **Female** | Prescription, n (%) | 59.032 (71.8) |
|  |  | NDPP, mean ± SD | 2,19 ± 1,70 |
|  | **Male** | Prescription, n (%) | 23.137 (28.2) |
|  |  | NDPP, mean ± SD | 2,06 ± 1,62 |
| **Age group** | **18-65 years** | Prescription, n (%) | 60.977 (74.2) |
|  |  | NDPP, mean ± SD | 1,98 ± 1,53 |
|  | **≥ 65 years** | Prescription, n (%) | 21.192 (25.8) |
|  |  | NDPP, mean ± SD | 2,64 ± 1,98 |
| **Total** | | Prescription, n (%) | 82.169 (100.0) |
|  |  | NDPP, mean ± SD | 2.15 ± 1.68 |
|  |  | Cost | $2,495,623.0 |

*ACPs: Antidepressant-containing prescriptions, NDPP: number of drugs per prescription*

**Supplementary Table 2.** Distribution of the most common diagnoses in prescriptions excluding psychiatric diagnoses.

| **Rank** | **Diagnose (ICD-10)** | **Prescription**  **n (%)** |
| --- | --- | --- |
| 1 | Diseases of the circulatory system (I) | 6.435 (26.8) |
| 2 | Diseases of the digestive system (K) | 3.696 (15.4) |
| 3 | Diseases of the respiratory system (J) | 3.015 (12.6) |
| 4 | Symptoms, signs and abnormal clinical and laboratory findings, not elsewhere classified (R) | 2.093 (8.7) |
| 5 | Factors influencing health status and contact with health services (Z) | 1.758 (7.3) |
| 6 | Diseases of the nervous system (G) | 1.576 (6.6) |
| 7 | Endocrine, nutritional and metabolic diseases (E) | 1.236 (5.1) |
| 8 | Diseases of the musculoskeletal system and connective tissue (M) | 1.048 (4.4) |
| 9 | Diseases of the skin and subcutaneous tissue (L) | 860 (3.6) |
| 10 | Diseases of the genitourinary system (N) | 799 (3.3) |
| **Other** | | 1.485 (6.2) |
| **Total** | | 24.001 (100.0) |
